# Rush Medical College

**Published:** 1856-10

**Authors:** 


*Rush Medical College.*

The regular annual course of medical instruction in this Institution commences on the first Monday in November. And we believe everything is in readiness for as full and valuable a course of instruction, in all the departments, as is to be found in any of the schools in our country. The hospitals are well-filled with patients, furnishing more material for clinical instruction than can be conveniently used; while the *material* and means of illustration in practical anatomy, surgery, &c. are limited only by the wants of the Faculty and the Class. Students have already begun to arrive (Oct. 3) for the purpose of attending to dissecting and clinical instruction one month before the regular term commences. The prospects are favorable for a full class.

